# Effects of pressure-controlled ventilation targeting end-inspiratory flow rate on pulmonary complications and inflammation levels in patients undergoing spinal surgery in the prone position: a randomized clinical trial

**DOI:** 10.1186/s12871-024-02439-3

**Published:** 2024-02-09

**Authors:** Na Wang, Yong Ye, Hui Lin, Tingting Sun, Yue Hu, Yuanhang Shu, Jing Tong, Yong Tao, Zeyu Zhao

**Affiliations:** 1 Department of Anesthesia Operation, The First People’s Hospital of Shuangliu District, Chengdu, West China Airport Hospital of Sichuan University), No. 120 of Chengbei Street, Dongsheng Town, Shuangliu District, Chengdu, 610200 Sichuan China; 2 Department of Anesthesiology, Sichuan Provincial Rehabilitation Hospital Affiliated Chengdu University of Traditional Chinese Medicine, No.81 of Bayi Road, Yongning Street, Wenjiang District, Chengdu, 611135 Sichuan China

**Keywords:** End-inspiratory flow rate, General anesthesia, Pressure-controlled ventilation, Prone position, Spinal surgery

## Abstract

**Background:**

This study assessed the impact of pressure-controlled ventilation (PCV) focusing on end-inspiratory flow rate on the incidence of postoperative pulmonary complications (PPCs) and inflammation levels in patients undergoing spinal surgery in the prone position.

**Methods:**

A total of 187 patients who underwent posterior spinal surgery were enrolled and randomly divided into 3 groups: 61 in the volume-controlled ventilation (VCV) group (group V), 62 in the PCV-volume-guaranteed (VG) group (group P_1_), and 64 in the PCV-VG end-expiratory zero flow rate group (group P_2_). Indicators including tidal volume (V_T_), peak airway pressure (P_peak_), and dynamic lung compliance (Cdyn) were recorded. The P_peak_, Cdyn, P_ET_CO2, and oxygenation index (PaO_2_/FiO_2_) after intubation (T_0_), after prone position (T_1_), 60 min after prone position (T_2_), and after supine position at the end of surgery (T_3_) of the three groups were collected.

**Results:**

In the within-group comparison, compared with T_0_, P_peak_ increased at T_1 − 2_ in groups V and P_1_ (*P* < 0.01), whereas it decreased at T_1 − 3_ in group P_2_ (*P <* 0.01). Cdyn decreased at T_1 − 2_ and PaO2/FiO2 increased at T_1 − 3_ in all three groups (*P* < 0.01), and PaO2/FiO2 increased at T_1 − 3_ (*P* < 0.01). Compared with group V, P_peak_ decreased at T_0 − 3_ in group P_1_ (*P* < 0.01) and at T_1 − 3_ in group P_2_ (*P* < 0.01), while Cdyn increased at T_0 − 3_ in groups P_1_ and P_2_ (*P* < 0.01). Compared with group P_1_, P_peak_ was elevated at T_0_ (*P* < 0.01) and decreased at T_1 − 3_ (*P* < 0.05), and Cdyn was elevated at T_0 − 3_ in group P_2_ (*P* < 0.01). The total incidence of PPCs in group P_2_ was lower than that in group V (*P* < 0.01). Compared with the preoperative period, serum interleukin 6 (IL-6) and C-reactive protein (CRP) levels were increased at 24 and 72 h after surgery in group V (*P* < 0.01), whereas that was increased at 24 h after surgery in group P_1_ and group P_2_ (*P* < 0.01). Compared with group V, serum IL-6 and CRP levels were reduced at 24 h after surgery in groups P_1_ and P_2_ (*P* < 0.01 or < 0.05).

**Conclusion:**

In patients undergoing spinal surgery in the prone position, PCV-VG targeting an end-inspiratory zero flow rate lowers the incidence of PPCs and inflammation levels.

## Background

Prone position is a standard postural positioning method for posterior spinal surgery under general anesthesia, which gives the surgeons a good operating field [1]. However, the prone position restricts lung expansion, manifesting as decreased lung compliance and increased airway resistance. Improper mechanical ventilation parameter settings can exacerbate lung injury, increase the levels of inflammatory factors in the body, and increase the incidence of postoperative pulmonary complications (PPCs) [2–4]. The ventilation techniques, such as volume-controlled ventilation (VCV) and pressure-controlled ventilation (PCV), are frequently used for patients under general anesthesia. Tidal volume (V_T_) is constant during VCV, ensuring the volume per minute of ventilation, but airway pressure varies greatly, which can result in pulmonary barotrauma [5]. During PCV, the pressure was held constant, and a higher initial inspiratory flow rate was used to achieve the desired pressure level. As the inspiratory process continues, an exponentially decreasing flow rate waveform is generated, causing the inhaled gas to be evenly diffused in the lung tissue in a laminar flow form until the end of inspiration, which lowers airway pressure and has a certain pulmonary protective effect [6, 7]. Pressure-controlled ventilation-volume guaranteed (PCV-VG) is a unique type of PCV ventilation mode that cannot effectively lower airway pressure or ensure uniform gas distribution if the end-inspiratory flow rate is not reduced to zero. At this point, by extending the inspiratory time, the inspiratory flow rate further decreases until it reaches zero, which is the zero-flow rate at the end of inhalation [8–10]. It is still unknown if this ventilation technique can lower PPCs and inflammation levels in patients who undergo spinal surgery in the prone position. To better direct clinical practice, we investigated the effect of the PCV mode on the incidence of PPCs and inflammatory levels in patients undergoing spinal surgery in the prone position.

## Methods

### General information

This prospective, randomized, controlled trial was approved by the ethics committee of West China Airport Hospital of Sichuan University (approval number: 2021-1-10), and informed consent forms were signed by the patients. The patients were divided into three groups according to the random number table method: 61 cases with the VCV mode and inspiration and expiration ratio of 1:2 (group V), 62 cases with the PCV-VG mode, and inspiration and expiration ratio of 1:2 (group P_1_); and 64 cases in the PCV-VG end-expiratory zero-flow rate group (group P_2_). The trial was registered in the Chinese Clinical Trial Registry just before recruiting the first participant, and the registration was approved on May 12, 2023, with registration number ChiCTR2300xxxxx. All procedures were conducted according to the CONSORT guidelines.

### Methods

Prior to surgery, the patients did not consume food or liquids for six hours. Electrocardiogram (ECG), blood pressure (BP), heart rate (HR), pulse oximetry (SpO_2_), and bispectral index (BIS) were monitored after the patients entered the operating room (Mindray Medical International, China). Anesthesia induction: intravenous injection of 0.03 mg/kg midazolam, 0.5 mg/kg sufentanil, 0.2 mg/kg cistercurium, and 1.5 mg/kg propofol. Mechanical ventilation was performed after endotracheal intubation (Drager Primus, Germany), with a V_T_ of 8 ml/kg, respiratory rate of 12–14 times/min, fresh gas flow of 2 L/min, inhaled oxygen concentration of 50%, positive end-expiratory pressure (PEEP) of 0 cmH_2_O (1 cmH_2_O = 0.098 kPa), and end-expiratory carbon dioxide partial pressure (P_ET_CO_2_) of 30–45 mmHg (1 mmHg = 0.133 kPa). The inspiration and expiration ratio of group V and P_1_ was 1:2, The inspiratory time setting in group P1 depended on the respiratory rate and I:E ratio, whereas group P2’s inspiratory time setting depends on the point at which EIFR reached zero in each patient. Anesthesia maintenance: intravenous infusion of 0.15 to 0.2 μg/ (kg min) remifentanil, inhalation of 2.0–8.0% desflurane, with BIS maintained at 40 to 55. Intraoperative hypotension (MAP < 20% of the basal value) was treated with intravenous injection of 6 mg ephedrine, and sinus bradycardia was treated with intravenous injection of atropine (0.5 mg). The operation was performed with the patient in the prone position. The patient was placed in the supine position after the operation, the anesthetic medications were stopped, endotracheal intubation was removed when the spontaneous breathing rate was ≥ 14 times per minute, the V_T_ was ≥ 300 mL, and the patient was then transported to the anesthesia recovery room for further observation and care.

### Observation indicators

Side-stream spirometry continuous airway pressure monitoring technology was used to track V_T_, peak airway pressure (P_peak_), and dynamic lung compliance (Cdyn). The P_peak_, Cdyn, P_ET_CO2, and oxygenation index (PaO_2_/FiO_2_) after intubation (T_0_), after prone position (T_1_), 60 min after prone position (T_2_), and after supine position at the end of surgery (T_3_) of the three groups were collected. The formula for calculating Cdyn is Cdyn = TV/ (PIP - PEEP). Operation time, anesthesia time, blood loss, blood infusion, and fluid volume were recorded. The occurrence of PPCs, including respiratory tract infection, respiratory failure, pleural effusion, atelectasis, pulmonary edema, pneumothorax, bronchospasm, acute lung injury, and hypoxemia within 72 h after surgery, was recorded. Hypoxemia is characterized by an oxygen saturation level < 90%. Before surgery, 2 ml of peripheral venous blood was extracted 24 and 72 h after surgery, and the levels of interleukin 6 (IL-6) and C-reactive protein (CRP) were measured by enzyme-linked immunosorbent assay.

### Statistical analysis

The sample size was calculated by comparing two independent sample rates using the MedSci Sample Size tools. The incidence of pulmonary complications after mechanical ventilation is approximately 40% [11]. It is estimated that the incidence rate of pulmonary complications after the use of zero flow rate at the end of inspiration is approximately 15% (α = 0.05, β = 0.2), the ratio of sample size between the two matched groups was 1, and the bilateral test results showed a loss of follow-up ratio of 0.2. The calculated sample size for each group was 58 cases, and at least 58 cases were selected for each group of enrolled cases. In practice, the sample sizes of the three groups were 61, 62, and 64, respectively, totaling 187 cases, which is consistent with the sufficient sample size.

SPSS25.0 software was used to analyze the data. In this study, the sample size of each group was greater than 50; therefore, the Kolmogorov-Smirnov test was used. The measurement data conforming to normal distribution are expressed as mean ± standard deviation ($\bar x \pm s$). Analysis of variance (ANOVA) for repeated measurement design data was used for within-group comparison, paired *t*-test was used for the comparison between any two means, and one-way ANOVA was used for comparison between groups. A chi-squared test or Fisher’s exact probability method was used to compare counting data. Multiple comparisons were performed using Bonferroni test. Statistical significance was set at *P* < 0.05.

## Results

**Fig. 1:**
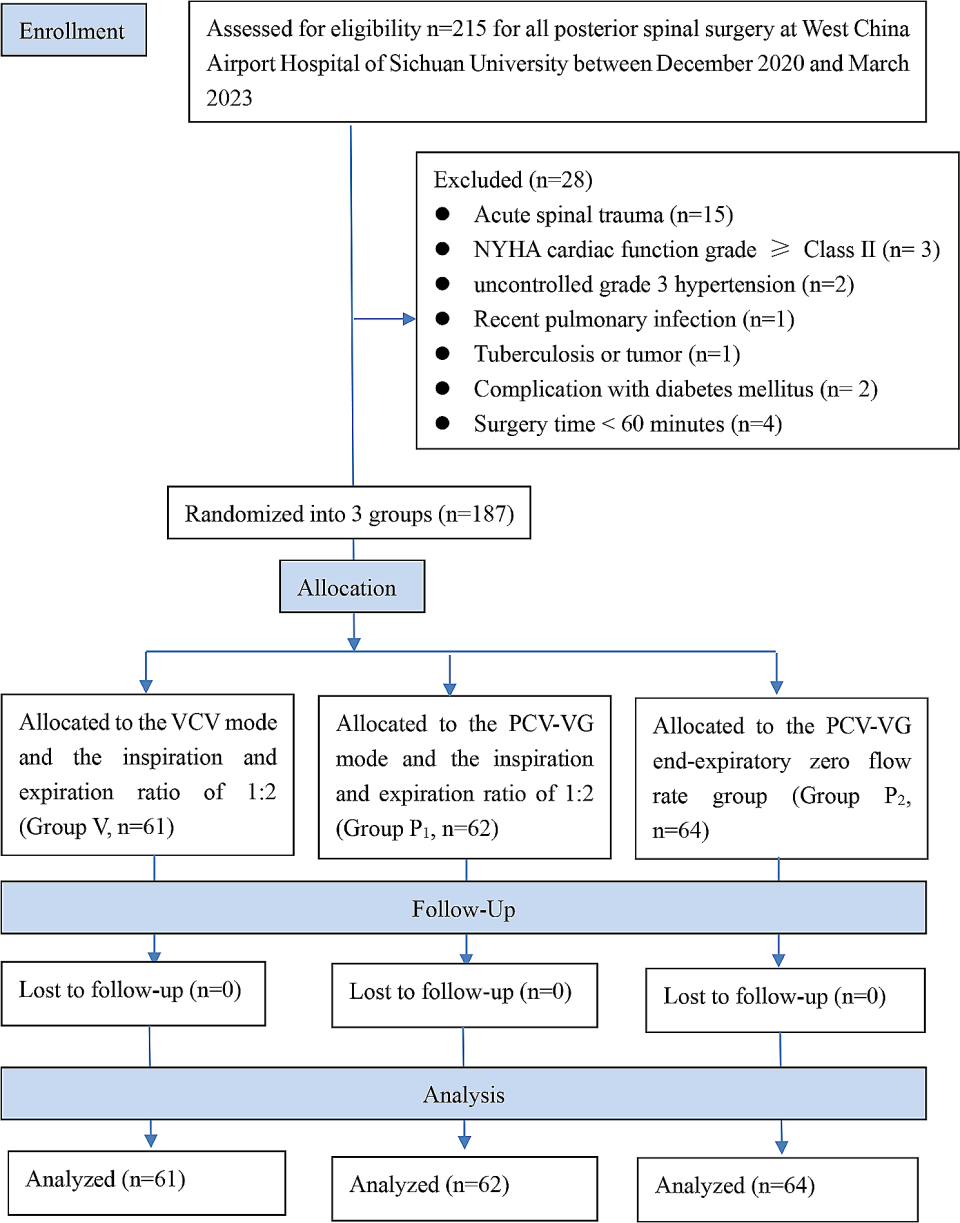
CONSORT diagram

A total of 215 patients who underwent posterior spinal surgery at the West China Airport Hospital of Sichuan University between December 2020 and March 2023 were selected. To exclude acute spinal trauma (*n* = 15), New York Heart Association (NYHA) cardiac function grade ≥ Class II (*n* = 3), uncontrolled grade 3 hypertension (*n* = 2), recent pulmonary infection (*n* = 1), tuberculosis or tumor (*n* = 1), complications of diabetes mellitus(*n* = 2), and surgery time < 60 min (*n* = 4). Ultimately, 187 patients were included in the study. Figure 1 shows the flow diagram.

### Comparison of general conditions and surgical categories of the three groups

There were no significant differences in age, gender composition, body mass index, ASA grade, and surgical category between the three groups (*P* > 0.05) (Table 1).

**Table 1:** Comparison of general conditions and surgical categories of the three groups

Indicators	Group V (*n* = 61)	Group P_1_ (*n* = 62)	Group P_2_ (*n* = 64)	F/χ^2^ value	*P* value
Age (years, $\bar x \pm s$)	45 ± 10	47 ± 11	48 ± 12	1.044	0.354
Male/female (n)	43/18	41/21	42/22	0.403	0.818
BMI (kg/m^2^, $\bar x \pm s$)	24.2 ± 2.9	24.1 ± 3.0	25.0 ± 3.1	1.543	0.216
ASA grade I/II (n)	23/28	26/36	24/40	0.328	0.849
Surgical categories (n)				1.599	0.999
Lumbar disc herniation	15	17	17		
Cervical spondylosis	17	13	15		
Spinal stenosis	12	12	13		
Lumbar spondylolisthesis	11	13	12		
Spinal tuberculosis	3	4	5		
Spinal tumor	3	3	2		

### Comparison of airway-related indexes in the three groups at different time points

There were differences in P_peak_, Cdyn, P_ET_CO_2_, and PaO_2_/FiO_2_ at different time points. P_peak_ and Cdyn were different among the three groups, and there were interactions between the Cdyn at different time points and different groups (*P* < 0.05). Further pairwise comparison and within-group comparison revealed that compared with T_0_, P_peak_ increased at T_1 − 2_ in group V and P_1_ (*P* < 0.01) and decreased at T_1 − 3_ in group P_2_ (*P* < 0.01). Cdyn decreased at T_1 − 2_ and PaO_2_/FiO_2_ increased at T_1 − 3_ in all three groups (*P* < 0.01), and PaO_2_/FiO_2_ increased at T_1 − 3_ (*P* < 0.01). Between-group comparisons revealed that compared with group V, P_peak_ decreased at T_0 − 3_ in group P_1_ (*P* < 0.01), and decreased at T_1 − 3_ in group P_2_ (*P* < 0.01), and Cdyn increased at T_0 − 3_ in group P_1_ and P_2_ (*P* < 0.01). Compared with group P_1_, P_peak_ was elevated at T_0_ (*P* < 0.01) and decreased at T_1 − 3_ (*P* < 0.05), and Cdyn was elevated at T_0 − 3_ in group P_2_ (*P* < 0.01). There were no significant differences between the remaining indicators (*P* > 0.05) (Table 2).

**Table 2:** Comparison of the airway-related indexes of the three groups at different time points

Groups	P_peak_(cmH2O)	Cdyn(ml/cmH_2_O)	P_ET_CO_2_(mmHg)	PaO_2_/FiO_2_(mmHg)
Group V (*n* = 61)				
T0	13.3 ± 1.5	39.0 ± 6.6	34.6 ± 4.6	198 ± 20
T1	15.4 ± 1.6 ^a^	33.7 ± 5.6 ^a^	35.1 ± 4.5	212 ± 23 ^b^
T2	15.5 ± 2.0 ^a^	33.8 ± 6.4 ^a^	35.2 ± 4.4	215 ± 18 ^b^
T3	13.3 ± 1.8	39.4 ± 7.3	34.3 ± 6.0	216 ± 21 ^b^
Group P_1_ (*n* = 62)				
T0	11.5 ± 1.0 ^c^	44.9 ± 8.7 ^c^	34.4 ± 4.7	201 ± 17
T1	13.5 ± 1.4 ^ac^	38.3 ± 7.7 ^ac^	34.8 ± 4.7	215 ± 20 ^b^
T2	13.7 ± 1.5 ^ac^	37.8 ± 7.7 ^ac^	35.0 ± 4.6	216 ± 23 ^b^
T3	11.6 ± 1.0 ^ac^	44.4 ± 8.6 ^c^	34.7 ± 4.5	218 ± 19 ^b^
Group P_2_ (*n* = 64)				
T0	13.3 ± 1.5 ^d^	51.2 ± 9.2 ^cd^	34.1 ± 4.9	204 ± 20
T1	12.4 ± 1.0 ^ace^	43.2 ± 8.3 ^acd^	34.7 ± 5.2	216 ± 18 ^b^
T2	12.5 ± 1.1 ^ace^	42.9 ± 8.6 ^acd^	34.8 ± 5.2	218 ± 22 ^b^
T3	10.2 ± 1.0 ^ace^	52.7 ± 10.6 ^cd^	34.0 ± 6.5	219 ± 17 ^b^
F_time_, P_time_	525.456,0.000	407.854,0.000	5.129,0.002	279.859,0.000
F_group_, P_group_	97.236,0.000	32.098,0.000	0.150,0.860	1.018,0.363
F _time*group_, P _time*group_	0.864,0.524	9.966,0.000	0.492,0.815	0.849,0.533

Note: Compared with T_0_, ^a^*P* < 0.01, ^b^*P* < 0.05; Compared with Group V, ^c^*P* < 0.01; Compared with Group P_1_, ^d^*P* < 0.01, ^e^*P* < 0.05. P_peak_: peak airway pressure, Cdyn: lung dynamic compliance, P_ET_CO_2_: end-expiratory carbon dioxide partial pressure, PaO_2_/FiO_2_: oxygenation index

### Comparison of anesthesia time, operation time, and intraoperative intake and output volume of the three groups

There were no significant differences between the three groups in terms of anesthesia time, operating time, intraoperative infusion volume, blood loss, urine output, or number of blood transfusions (*P* > 0.05) (Table 3).

**Table 3:** Comparison of the anesthesia time, operation time, and intraoperative intake and output volume of the three groups

Group	n	Anesthesia time (min, $\bar x \pm s$)	Operation time (min, $\bar x \pm s$)	Infusion volume (mL, $\bar x \pm s$)	Blood loss (mL, $\bar x \pm s$)	Urine output (mL, $\bar x \pm s$)	Number of blood transfusion cases [n (%)]
Group V	61	189 ± 44	134 ± 41	1149 ± 330	438 ± 208	275 ± 144	7(11.48)
Group P_1_	62	195 ± 47	140 ± 43	1219 ± 360	473 ± 164	312 ± 136	5(8.06)
Group P_2_	64	186 ± 54	131 ± 47	1155 ± 378	458 ± 161	298 ± 138	6(9.38)
*F/χ*^*2*^ value		0.512	0.647	0.493	0.590	1.161	0.418
*P* value		0.600	0.525	0.611	0.555	0.315	0.811

### Comparison of the incidence of PPCs and hypoxemia in the three groups

**Fig. 2:**
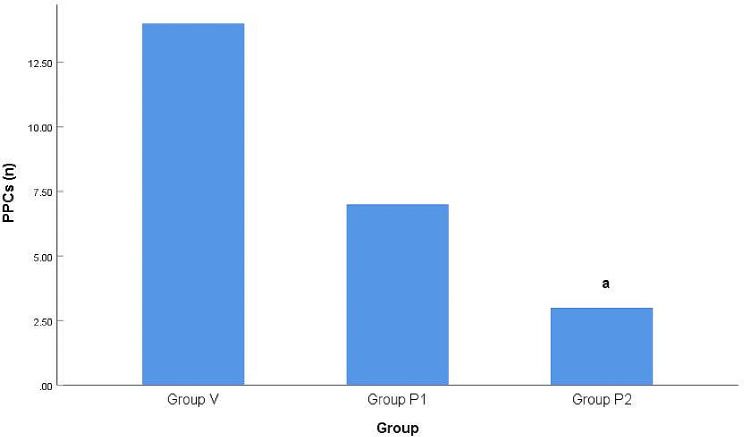
Comparison of the incidence of PPCs Note: Compared with Group V, ^a^*P* < 0.01

**Fig. 3:**
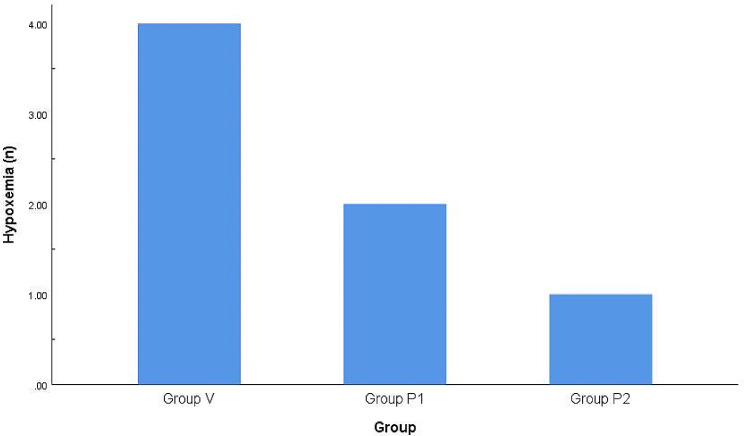
Comparison of the incidence of hypoxemia

The total incidence of PPCs among the three groups was significantly different (*P* < 0.01), and further between-group comparison revealed that the total incidence of PPCs in group P_2_ was lower than that in group V (*P* < 0.01). There were no significant differences in the incidence of hypoxemia 72 h after surgery among the three groups (*P* > 0.05) (Figs. 2 and 3).

### Comparison of serum IL-6 and CRP levels at different points in the three groups

**Fig. 4:**
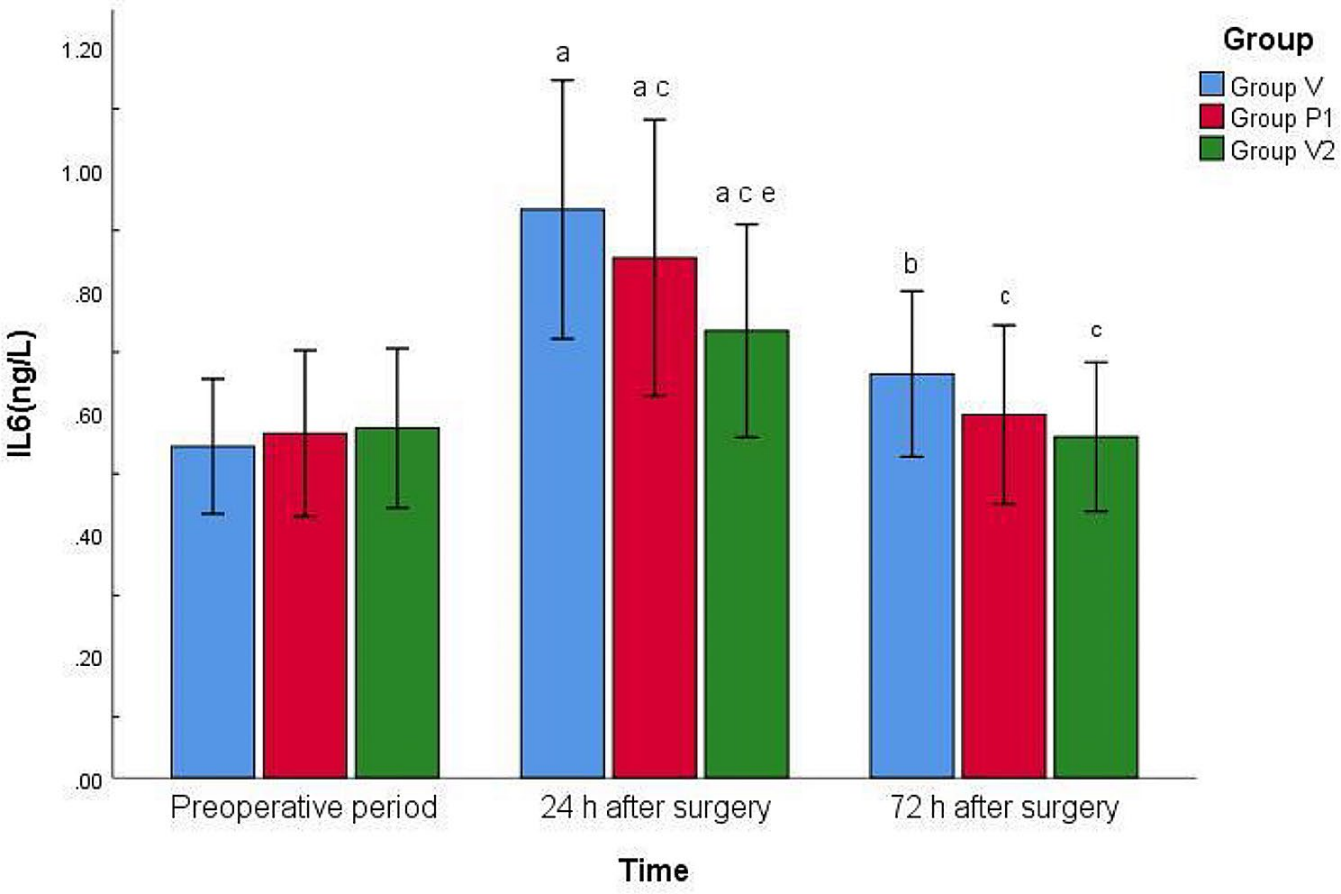
Comparison of serum IL-6 levels of the three groups at different points Note: Compared with preoperative period, ^a^*P* < 0.01, ^b^*P* < 0.05; Compared with Group V, ^c^*P* < 0.01, ^d^*P* < 0.05; Compared with Group P_1_, ^e^*P* < 0.05

**Fig. 5:**
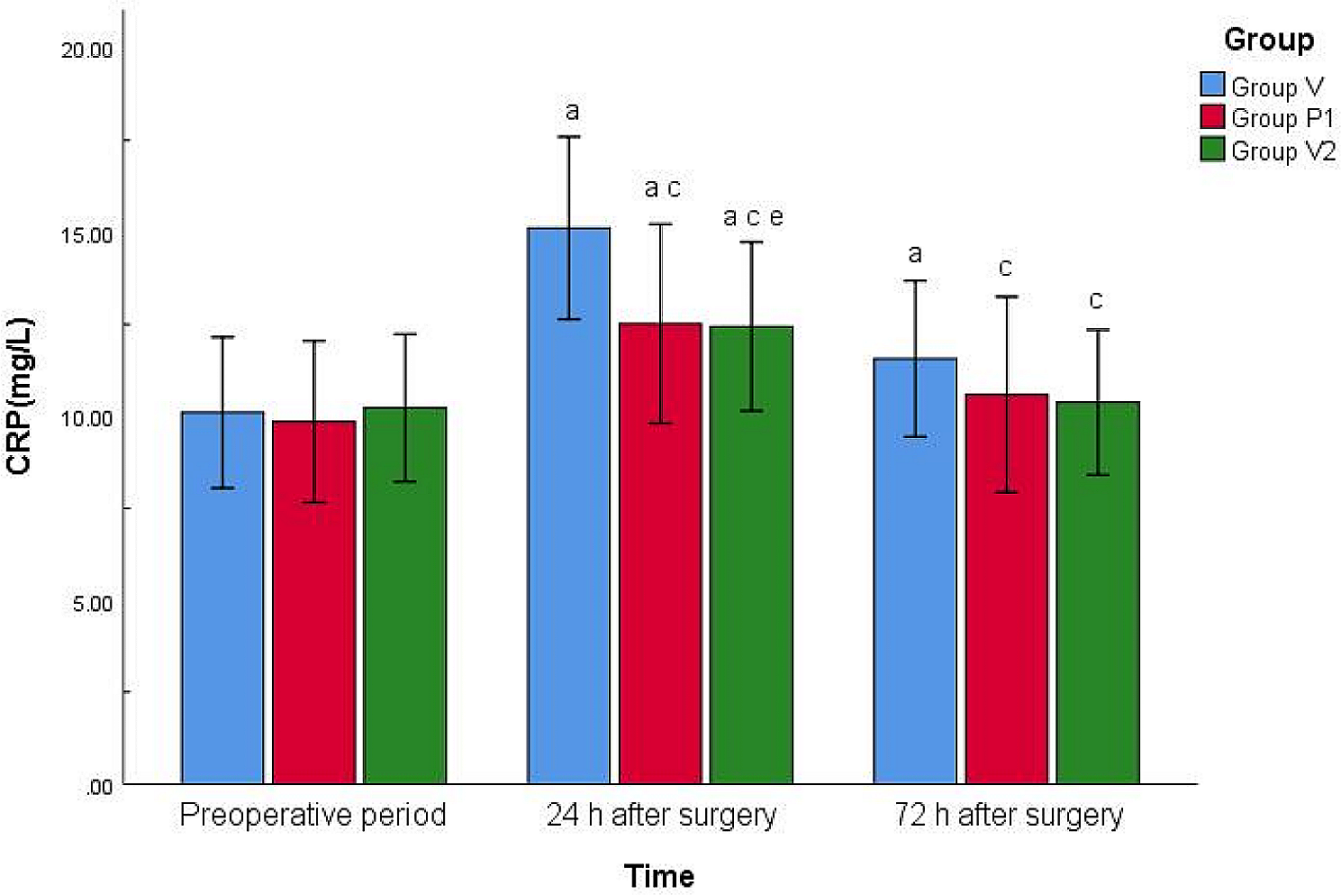
Comparison of serum CRP levels of the three groups at different points Note: Compared with preoperative period, ^a^*P* < 0.01, ^b^*P* < 0.05; Compared with Group V, ^c^*P* < 0.01, ^d^*P* < 0.05; Compared with Group P_1_, ^e^*P* < 0.05

There were no significant differences in preoperative serum IL-6 and CRP levels among the three groups (*P* > 0.05). Compared with the preoperative period, the serum IL-6 and CRP levels increased at 24 and 72 h after surgery in group V (*P* < 0.01), whereas the serum IL-6 and CRP levels increased at 24 h after surgery in groups P_1_ and P_2_ (*P* < 0.01). Compared with group V, serum IL-6 and CRP levels were reduced at 24 h after surgery in groups P_1_ and P_2_ (*P* < 0.01 or < 0.05). Compared with group P_1_, the serum IL-6 and CRP levels were reduced 24 h after surgery in group P_2_ (*P* < 0.05). There were no significant differences between the remaining indicators (*P* > 0.05) (Figs. 4 and 5).

## Discussion

The prone position is common in general anesthesia surgery; however, surgery in the prone position restricts chest wall movement, leading to decreased compliance and increased airway pressure. Improper mechanical ventilation can exacerbate lung injury and increase the incidence of PPCs, including respiratory infections, respiratory failure, pleural effusion, atelectasis, pulmonary edema, pneumothorax, and acute lung injury. Commonly used ventilation modes during general anesthesia include VCV and PCV. Although the VCV mode can meet tidal volume and minute ventilation requirements, it exhibits large fluctuations in airway pressure, increasing the risk of barotrauma. The PCV mode can generate an exponentially decreasing flow waveform, evenly distribute inhaled gas throughout the lung tissue, promote the expansion of distal alveoli, and lower airway pressure. In PCV mode, inhalation begins with a higher flow rate to reach the preset pressure level, followed by an exponentially decreasing flow rate until the end of inhalation. If the flow rate at the end of inhalation does not drop to “0,” the inhalation time may be prolonged, and the flow rate will further decrease until it reaches “0,” which is known as the end-inspiratory zero flow rate. In this study, we randomly assigned patients to the VCV group, PCV-VG group with constant inspiratory time, and PCV-VG group with end-expiratory zero flow rate. The V_T_ value was set according to the standard body weight. However, the absence of a pure PCV mode group is based on the fact that under pure PCV mode, the VT value is difficult to meet the set requirements with changes in the thoracic cage, lung compliance, and airway pressure after constant pressure [12]. In contrast, in the PCV-VG mode, the peak inspiratory flow rate was automatically adjusted, ensuring consistency of the V_T_ value between group P_1_ with end-expiratory non-zero flow rate, group P_2_ with end-expiratory zero flow rate, and group V with fixed volume mode.

Based on the results of this study, patients who underwent spinal surgery in the prone position experienced a lower incidence of PPCs when PCV-VG targeting end-expiratory zero flow rate was used. During prone mechanical ventilation, the anterior thoracic wall and abdomen are compressed, passive expansion is restricted, thoracic and lung compliance is reduced, and airway pressure is increased. Therefore, ventilation in standard VCV mode may aggravate lung injury and increase the prevalence of PPCs [13, 14]. This study was designed to determine whether the PCV-VG mode that targets end-inspiratory flow rate is helpful in enhancing patient outcomes after spinal surgery in the prone position. The results revealed that the respiratory mechanics-related parameters for patients undergoing spinal surgery in the prone position could be considerably enhanced using the PCV mode targeting the end-inspiratory flow rate. Compared with group V, P_peak_ decreased and Cdyn increased in groups P_1_ and P_2_, respectively. In addition, P_peak_ decreased and Cdyn increased significantly in group P_2_, which ultimately improved respiratory mechanics-related indicators and reduced the incidence of PPCs. This is consistent with previous studies [15]. P_peak_ represents preset airway pressure with PCV ventilation. This ventilation strategy can limit the intra-alveolar pressure and more evenly distribute gas, both of which have a considerable positive impact on the oxygenation levels and lung injury severity in patients [16]. As the PCV ventilation mode is achieved by adjusting the inspiratory pressure level, the initial pressure is high, and V_T_ is also influenced by thoracic and lung compliance as well as airway resistance [17]. PCV-VG, a special type of PCV, involves initiating inhalation at a predetermined pressure level achieved through a higher initial flow rate. Subsequently, the inhalation flow rate progressively decreases exponentially with inhalation time until the conclusion of inhalation [18, 19]. Due to increased V_T_, if the inspiratory velocity does not decrease to the “0” point at the end of inhalation, it may result in a longer inspiratory period and excessive alveolar inflation, whereas a shorter inspiratory period and a lower V_T_ may result in atelectasis [15]. To more accurately regulate the PCV ventilation strategy in the prone position, the end-inspiratory flow rate is adjusted to “0” by adjusting the inspiratory time, which is, the end-inspiratory zero flow rate, which not only effectively avoids hyperventilation-induced alveolar expansion, but also prevents atelectasis caused by hypoventilation.

The results of this study also demonstrated how PCV-VG, targeting the end-expiratory zero flow rate, can lower the inflammation level in patients 24 h after spinal surgery in the prone position. It is widely known that acute trauma can lead to an increase in inflammatory factors such as IL-6 [12], whereas CRP is a naturally occurring immunological protein whose plasma concentration rapidly rises with acute tissue injury [20]. As a result, patients with acute spinal trauma were excluded in this study. Acute trauma can directly change cytokine activity and considerably elevate the levels of pro-inflammatory cytokines IL-6 and CRP, which can lead to an excessive inflammatory response in the body [21, 22]. In this study, the preoperative levels of serum IL-6 and CRP in all three groups were within the normal range. Serum IL-6 and CRP levels increased in all three ventilation modes 24 h after surgery, whereas the levels of serum IL-6 and CRP in the VCV ventilation mode continued to increase 72 h post-surgery. Serum IL-6 and CRP levels were considerably lower in patients with PCV-VG mode targeting end-expiratory zero flow rate 24 h after surgery than in those with VCV ventilation mode. This implies that prolonged improper respiratory parameter setting during prone mechanical ventilation can exacerbate lung injury, whereas the PCV-VG mode targeting end-expiratory zero flow rate can reduce the severity of lung injury. However, a systematic review showed that serum IL-6 levels increased within 1–3 days after surgery and persisted for 2 weeks, while CRP typically increased within 48 h [23]. The results of this study regarding these biomarkers, although statistically significant, may not be clinically significant. In addition, the subjects of this study were patients with chronic spinal disorders, and those with acute spinal trauma, which could affect the level of serum inflammatory factors, were excluded. The results showed that the pressure-controlled ventilation mode targeting zero flow rate at the end of inspiration can reduce the incidence of postoperative pulmonary complications and the level of inflammation in patients undergoing spinal surgery in the prone position, which is consistent with the results of a previous study [24]. 

Although this study showed that the PCV mode targeting zero flow rate at the end of inspiration can significantly improve the incidence of postoperative pulmonary complications and the level of inflammation in patients undergoing spinal surgery in the prone position, it still has some limitations, such as small sample size. Additionally, this type of study has higher requirements for the anesthesia machine, requiring the equipment to have precise adjustments for flow waveform monitoring and inspiration time under the PCV mode. Future studies should expand the sample size and optimize the anesthesia equipment to further validate this conclusion and enhance patient safety.

## Conclusions

In conclusion, implementation of PCV mode targeting end-expiratory zero flow rate in patients undergoing spinal surgery in the prone position can potentially reduce postoperative pulmonary complications and inflammation levels. These findings suggest a practical strategy to enhance patient outcomes in this specific context. Future research should focus on refining the methodology and exploring broader applications of this ventilation strategy across various surgical procedures performed in the prone position.

## Abbreviations

ASA: American Society of Anesthesiologists physical status
BIS: Bispectral index
BP: Blood pressure
Cdyn: Dynamic lung compliance
CRP: C-reactive protein
ECG: Electrocardiogram
HR: Heart rate
IL-6: Interleukin-6
MAP: Mean Arterial pressure
P_ET_CO_2_: End-tidal carbon dioxide partial pressure
P_peak_: Peak airway pressure
PaO_2_/ FiO_2_: Oxygenation index
PCV: PressureControlled ventilation
PCV VG: Pressure-controlled ventilation-volume guaranteed
PEEP: Positive end expiratory pressure
PPCs: Postoperative pulmonary complications
SpO_2_: Pulse oximetry
V_T_: Tidal volume
VCV: Volumecontrolled ventilation
